# Fingolimod promotes blood–nerve barrier properties in vitro

**DOI:** 10.1002/brb3.924

**Published:** 2018-03-25

**Authors:** Hideaki Nishihara, Toshihiko Maeda, Yasuteru Sano, Maho Ueno, Nana Okamoto, Yukio Takeshita, Fumitaka Shimizu, Michiaki Koga, Takashi Kanda

**Affiliations:** ^1^ Department of Neurology and Clinical Neuroscience Yamaguchi University Graduate School of Medicine Ube Japan

**Keywords:** Blood–nerve barrier, chronic inflammatory demyelinating polyneuropathy, fingolimod, S1P

## Abstract

**Objective:**

The main effect of fingolimod is thought to be functional antagonism of lymphocytic S1P1 receptors and the prevention of lymphocyte egress from lymphoid tissues, thereby reducing lymphocyte infiltration into the nervous system. However, a growing number of reports suggest that fingolimod also has a direct effect on several cell types in the nervous system. Although we previously reported that fingolimod enhances blood–brain barrier (BBB) functions, there have been no investigations regarding the blood–nerve barrier (BNB). In this study, we examine how fingolimod affects the BNB.

**Methods:**

An immortalized human peripheral nerve microvascular endothelial cell line (HPnMEC) was used to evaluate BNB barrier properties. We examined tight junction proteins and barrier functions of HPnMECs in conditioned medium with or without fingolimod‐phosphate and blood sera from patients with typical chronic inflammatory demyelinating polyneuropathy (CIDP).

**Results:**

Incubation with fingolimod‐phosphate increased levels of claudin‐5 mRNA and protein as well as TEER values in HPnMECs. Conversely, typical CIDP sera decreased claudin‐5 mRNA/protein levels and TEER values in HPnMECs; however, pretreatment with fingolimod‐phosphate inhibited the effects of the typical CIDP sera.

**Conclusions:**

Fingolimod‐phosphate directly modifies the BNB and enhances barrier properties. This mechanism may be a viable therapeutic target for CIDP, and fingolimod may be useful in patients with typical CIDP who have severe barrier disruption.

## 1 INTRODUCTION

The main effect of fingolimod is thought to be the functional antagonism of the S1P1 receptor. However, there has also been some focus on its multifunctional effects on several cell types such as astrocytes, oligodendrocytes, neurons, and microglia (Soliven, Miron, & Chun, 2011). We recently reported that fingolimod‐phosphate, an active metabolite of fingolimod phosphorylated in vivo by sphingosine kinases, enhances the barrier properties of the blood–brain barrier (BBB) by upregulating claudin‐5 expression (Nishihara et al., 2015). While this result suggested that fingolimod‐phosphate directly affects the endothelial cells of the BBB and alters their properties, its effects on the blood–nerve barrier (BNB) have yet to be examined. Our previous study also demonstrated that sera from patients with chronic inflammatory demyelinating polyneuropathy (CIDP) could disrupt the BNB (Shimizu, Sawai, et al., 2014). In this study, we examined the effects of fingolimod on human peripheral nerve microvascular endothelial cells (HPnMECs) and evaluated whether fingolimod‐phosphate could block the disruption of the BNB caused by exposure to sera from patients with CIDP.

## 2 MATERIALS AND METHODS

### 2.1 Sera

The ethics committee of the Medical Faculty, Yamaguchi University, approved this study and written informed consent was obtained from each participant. The ethics committee of Yamaguchi University also approved this consent procedure. Serum was collected from 10 typical patients with CIDP characterized by the following: chronically progressive or recurrent symmetric proximal and distal weakness and sensory dysfunction of all extremities developing over at least 2 months, in the initial progressive phase of the disease or at relapse, diagnosed at Yamaguchi University Hospital. All patients met the clinical criteria based on the EFNS/PNS guidelines (Van den Bergh et al., 2010). The inclusion criteria were definite or probable CIDP. None of the patients had a history of previous immunomodulatory treatment, such as corticosteroids, intravenous immunoglobulin (IVIg) treatment, or plasma exchange in the 6 months prior to the collection of serum. Sera obtained from 10 healthy subjects served as normal controls. The blood samples were stored at −80°C until use. All sera were inactivated at 56°C for 30 min immediately prior to use.

### 2.2 Reagents

The culture medium used for the endothelial cells has been previously described (Sano et al., 2010). Polyclonal anti‐claudin‐5 and anti‐occludin antibodies were purchased from Zymed (San Francisco, CA, USA). Polyclonal antibeta tubulin was obtained from Santa Cruz Biotechnology (Santa Cruz, CA, USA). Fingolimod and fingolimod‐phosphate were provided by Mitsubishi Tanabe Pharma (Osaka, Japan). S1P was purchased from Avanti Polar Lipids (Avanti Polar Lipids Inc., AL, USA).

### 2.3 Cell culture and treatment with fingolimod, fingolimod‐phosphate, or sera

Immortalized human HPnMECs were generated as previously described (Abe et al., 2012). The cells were cultured in conditioned medium with/without fingolimod or fingolimod‐phosphate in a CO_2_ incubator at 37°C for 12 hr before total mRNA was extracted. After an additional 24 hr, total protein was obtained, and TEER values and permeability were measured. For sera treatments, cells were cultured with conditioned medium containing 10% individual serum from either patients with CIDP or healthy controls.

### 2.4 Reverse transcription‐polymerase chain reaction (RT‐PCR) analysis

The protocol for the RT‐PCR analysis was previously described (Sano et al., 2010). The following human primer pairs were used as follows: forward primer (5′‐TGCGGGAAGGGAGTATGTTT ‐3′) and reverse primer (5′‐CGATGGCGAGGAGACTGAAC ‐3′) for S1P1(van Doorn et al., 2012); forward primer (5′‐ TCTCTACGCCAAGCATTATGTGC ‐3′) and reverse primer (5′‐ TGGCCAACAGGATGATGGA ‐3′) for S1P2(van Doorn et al., 2012); forward primer (5′‐ TGCAGCTTCATCGTCTTGGAG ‐3′) and reverse primer (5′‐ GCCAATGAAAAAGTACATGCGG ‐3′) for S1P3 (van Doorn et al., 2012); forward primer (5′‐ CTGCTCTTCACCGCCCTGGC ‐3′) and reverse primer (5′‐ GAAGCCGTAGACGCGGCTGG ‐3′) for S1P4 (Cordts et al., 2011); forward primer (5′‐ GTGAGGTGGGAGCCATAGAA ‐3′) and reverse primer (5′‐ TTGGCTGAGTCTCCCAGAGT ‐3′) for S1P5 (Cordts et al., 2011).

### 2.5 Quantitative real‐time PCR analysis

We used the primer sequences and performed PCR analysis as previously described (Shimizu et al., 2012). G3PDH was used as an internal standard. The Stratagene Mx3005P instrument (Stratagene, Cedar Creek, Texas, USA) was used to perform the quantitative real‐time PCR analyses, and the relative quantities were calculated according to the formula Rv=RGene/RG3PDH using a software program, as previously described (Tasaki et al., 2014).

### 2.6 Western blot analysis

We used the same methodology as described in a previous study (Nishihara et al., 2015). For quantification, each band density was corrected to that of the antibeta tubulin band density using the Quantity One software program (Bio‐Rad, Hercules, CA), and changes in the expression of tight junction proteins, including claudin‐5 and occludin, in the HPnMECs were examined.

### 2.7 Immunocytochemistry

The methodology was previously described (Nishihara et al., 2016; Sano et al., 2010). A polyclonal rabbit anti‐claudin‐5 antibody (1:500 dilution; Zymed) was used as the primary antibody, and a FITC‐conjugated anti‐rabbit antibody (1:3,000 dilution; Thermo Fisher Scientific) was used as the secondary antibody. Fluorescence was detected using a fluorescence microscope (BZ‐9000; Keyence, Osaka, Japan).

### 2.8 Transendothelial electrical resistance studies

The TEER values of the cell layers were measured using a Millicell electrical resistance apparatus (Endohm‐6 and EVOM, World Precision Instruments, Sarasota, FL, USA) as previously described (Sano et al., 2010). For the measurement of TEER values, conditioned media (containing serum from either healthy controls or patients with CIDP and/or fingolimod‐phosphate) were randomly assigned prior to measurement, and the examiner was not informed of the treatment conditions of each sample. Changes in TEER values were repeatedly measured on different days using the same samples in triplicate.

### 2.9 Permeability studies

The HPnMECs were grown to confluence on 24‐well transwell cell culture inserts (0.4 μm pore size) at 37°C. Solute paracellular permeability was assessed using 10 kDa dextran‐conjugated FITC (1 mg/ml; Sigma‐Aldrich), and fluorescence in the lower chamber was measured using an MX3000P instrument (Stratagene) as previously described (Shimizu, Omoto, et al., 2014).

### 2.10 Data analysis

All comparisons of the median values between the groups were made using the Mann–Whitney *U* test or paired Student's *t* test using the GraphPad Prism software program (GraphPad, La Jolla, CA), and a two‐sided *p* value <.05 was considered to be statistically significant.

## 3 RESULTS

### 3.1 HPnMECs express the S1P1 and S1P2 receptors

**Figure 1:**
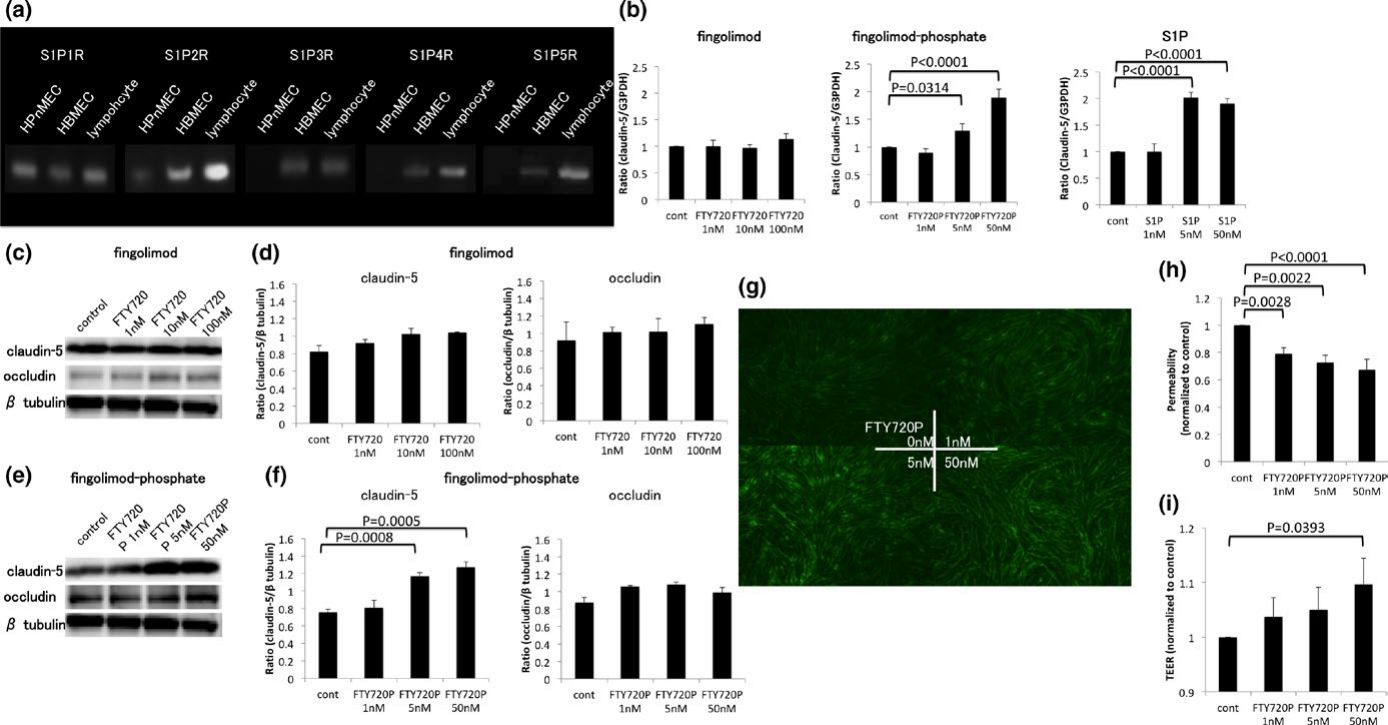
Effects of fingolimod and fingolimod‐phosphate on HPnMECs. (a) The expression of S1P receptors in HPnMECs was examined using RT‐PCR. The HPnMECs expressed S1P1 and S1P2 receptors. (b) The effects of fingolimod, fingolimod‐phosphate, and S1P on mRNA expression of tight junction proteins. Fingolimod‐phosphate and S1P increased the expression levels of claudin‐5. (c, e) The effect of fingolimod and fingolimod‐phosphate on tight junction protein levels. (d, f) The bar graphs reflect the combined densitometry data for each independent experiment. (g) Immunocytochemistry for the expression of claudin‐5 in HPnMECs. Treatment with fingolimod‐phosphate increased claudin‐5 protein levels (h). (i) Functional analysis of the blood–nerve barrier after fingolimod‐phosphate treatment. Fingolimod‐phosphate increased the TEER values and decreased the permeability of HPnMECs. FTY720 = fingolimod, FTY720P = fingolimod‐phosphate

We examined which S1P receptors were expressed by the HPnMECs using RT‐PCR. HPnMECs expressed the S1P1 and S1P2 receptors (Figure 1a), while HBMECs expressed the S1P1, S1P2, S1P3, S1P4, and S1P5 receptors. Lymphocytes were used as controls.

### 3.2 Fingolimod‐phosphate increases expression of claudin‐5 mRNA and protein in HPnMECs

The expression of mRNAs for tight junction proteins was examined by quantitative real‐time PCR. Fingolimod‐phosphate and S1P significantly upregulated the level of claudin‐5 mRNA (Figure 1b), although it did not affect the level of occludin (data not shown). In contrast, incubation with fingolimod did not alter the expression of tight junction protein mRNA in the HPnMECs (Figure 1b). Furthermore, the level of claudin‐5 protein was elevated after exposure to fingolimod‐phosphate as indicated by Western blot analysis (Figure 1e,f) and immunocytochemistry (Figure 1g). Fingolimod, however, did not have this effect on HPnMECs (Figure 1c,d).

### 3.3 Fingolimod‐phosphate enhances the barrier function of the BNB

Barrier functions of the BNB were evaluated based on TEER values and permeability. Fingolimod‐phosphate caused a dose‐dependent decrease in endothelial cell permeability (Figure 1h). In addition, high‐dose fingolimod‐phosphate increased the TEER values in HPnMECs (Figure 1i).

### 3.4 Pretreatment with fingolimod‐phosphate prevents the barrier disruption caused by CIDP sera

**Figure 2:**
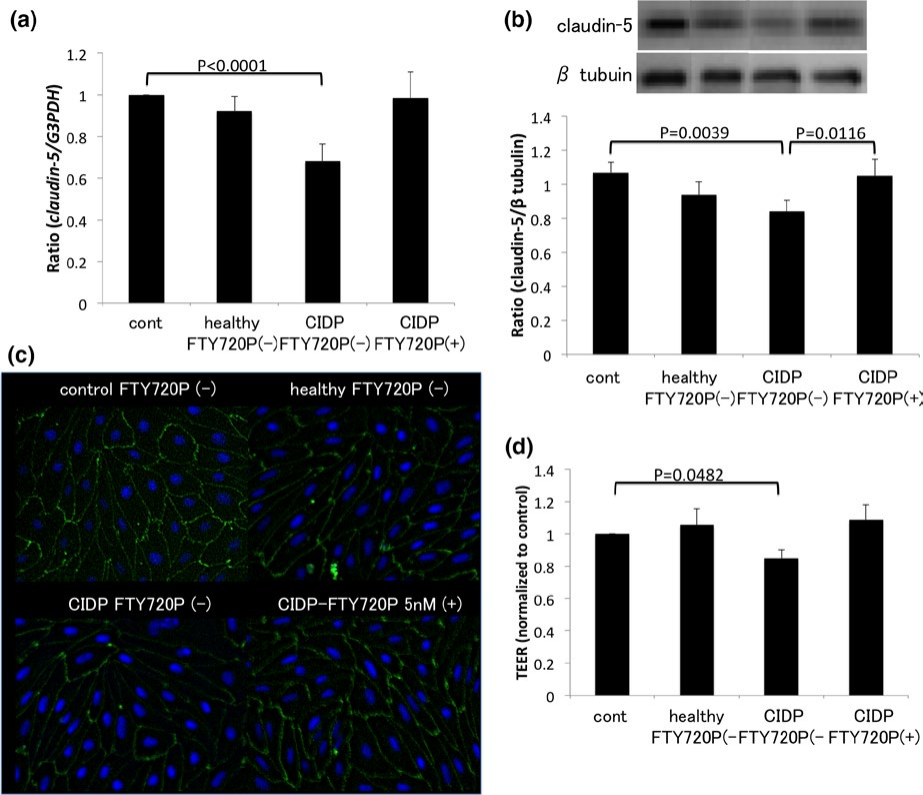
Effects of fingolimod‐phosphate pretreatment on HPnMECs in the presence of CIDP sera. (a) The mRNA expression levels of *claudin‐5* were evaluated using qPCR. Exposure to CIDP sera downregulated the expression levels of *claudin‐5*, whereas pretreatment with fingolimod‐phosphate prevented those effects (b). (c) Protein levels of claudin‐5 were examined by Western blot (b) and immunocytochemistry (c). (b) Each bar graph reflects the combined densitometry data for the independent experiments (mean ± *SEM*; healthy *n* = 10, CIDP *n* = 10, CIDP‐FTY720P *n* = 10). Sera from patients with CIDP decreased claudin‐5 protein levels. However, pretreatment with fingolimod‐phosphate blocked the effects of CIDP sera on tight junction proteins. (d) TEER values in HPnMECs. Although CIDP sera decreased the TEER values in HPnMECs, pretreatment with fingolimod‐phosphate protected the BNB from the disrupting factors in the CIDP sera

Figure 2 shows the effects of sera from patients with CIDP and from healthy controls, as well as the effects of fingolimod‐phosphate pretreatment. The level of claudin‐5 mRNA expression and protein in HPnMECs was examined using quantitative real‐time PCR and Western blot analyses. Sera from patients with CIDP significantly decreased claudin‐5 mRNA expression (Figure 2a) and protein levels (Figure 2b,c). However, pretreatment with 5 nmol/L fingolimod‐phosphate prevented these effects. In addition, treatment with CIDP sera decreased the TEER values in HPnMECs, and this effect was blocked by pretreatment with fingolimod‐phosphate (Figure 2d).

## 4 DISCUSSION

Fingolimod is thought to provide therapeutic effects to MS patients by preventing the egress of lymphocytes from the lymph nodes, thus reducing the degree of infiltration into the CNS. In regard to the peripheral nervous system (PNS), a previous report showed that fingolimod ameliorates disease conditions in animal models of experimental autoimmune neuritis (Zhang, Zhang, Fauser, & Schluesener, 2008, 2009; Zhang, Zhang, & Schluesener, 2009) and spontaneous autoimmune polyneuropathy (Kim et al., 2009). These studies demonstrated that the number of infiltrating Th17 cells was decreased in peripheral nerves, whereas the number was increased in lymph nodes, suggesting that the therapeutic effect of fingolimod in experimental autoimmune neuritis has a similar mechanism as in MS patients.

An increasing number of studies have stressed that fingolimod also has a direct effect on several cells within the CNS, including astrocytes, oligodendrocytes, microglia, and neurons (Miron, Schubart, & Antel, 2008; Soliven et al., 2011). As for endothelial cells, our previous study revealed direct BBB‐modulating effects which included the enhancement of the barrier properties of the BBB by upregulation of claudin‐5 expression and inhibition of the increase in VCAM‐1 levels in BMECs induced by MS sera (Nishihara et al., 2015). Other laboratories have also shown that S1P receptor signaling reduces the cell death resulting from inflammatory cytokines (Spampinato et al., 2015). These reports indicated that fingolimod directly affects endothelial cells which might contribute to its efficacy in the treatment of MS. However, little is known about the efficacy of fingolimod on the endothelial cells that comprise the BNB. In our present report, we first demonstrated that HPnMECs, which comprise the BNB, express S1P1 and S1P2 receptors. This suggested that fingolimod could exert its effects via the S1P1 receptor, as fingolimod is known to act on the S1P1, S1P3, S1P4, and S1P5 receptors.

There are many phenotypic variants of CIDP suggesting that it may not be a discrete disease and that the pathogenic mechanism of CIDP is heterogeneous (Mathey et al., 2015). Disruption of the BNB is considered to be a key step in the development of autoimmune diseases of the PNS (Kanda, 2013; Kanda, Numata, & Mizusawa, 2004; Kanda, Yamawaki, & Mizusawa, 2003). Our previous data suggested that the severity and pattern of BNB breakdown differ depending on the CIDP phenotype. For example, sera obtained from typical patients with CIDP more prominently reduced claudin‐5 protein levels and TEER values in HPnMECs compared to sera obtained from patients with multifocal acquired demyelinating sensory and motor neuropathy (MADSAM) or distal acquired demyelinating symmetric neuropathy (DADS) (Shimizu, Sawai, et al., 2014). In this report, we reconfirmed that CIDP sera disrupt the BNB via downregulation of claudin‐5. In addition, pretreatment with 5 nmol/L fingolimod‐phosphate, equivalent to the clinical dosage, prevented the BNB‐disrupting effects of CIDP sera. Taken together, fingolimod‐phosphate directly interacts with the BNB‐comprising endothelial cells, and the BNB might be a therapeutic target for patients with CIDP especially when the BNB is severely affected.

In conclusion, the present findings demonstrate that pretreatment with fingolimod‐phosphate enhances the barrier properties of the BNB by upregulating claudin‐5 expression in HPnMECs. These results suggest that fingolimod may prevent humeral factors from crossing the BNB into the PNS via its direct effects on HPnMECs. The direct BNB‐modulating effects of fingolimod may represent a possible novel venue for treating patients with CIDP.

## CONFLICT OF INTEREST

None declared.
